# Passive Aβ Immunotherapy: Current Achievements and Future Perspectives

**DOI:** 10.3390/molecules23051068

**Published:** 2018-05-03

**Authors:** Stephan Schilling, Jens-Ulrich Rahfeld, Inge Lues, Cynthia A. Lemere

**Affiliations:** 1 Fraunhofer Institute for Cell Therapy and Immunology, Department for Drug Design and Target Validation, 06120 Halle (Saale), Germany; Jens-Ulrich.Rahfeld@izi.fraunhofer.de; 2 Probiodrug AG, 06120 Halle (Saale), Germany; inge.lues@probiodrug.de; 3 Ann Romney Center for Neurologic Diseases, Brigham and Womens’s Hospital, Harvard Medical School, Boston, MA 02116, USA; clemere@bwh.harvard.edu

**Keywords:** amyloid-β, monoclonal antibodies, posttranslational modifications, drug development

## Abstract

Passive immunotherapy has emerged as a very promising approach for the treatment of Alzheimer’s disease and other neurodegenerative disorders, which are characterized by the misfolding and deposition of amyloid peptides. On the basis of the amyloid hypothesis, the majority of antibodies in clinical development are directed against amyloid β (Aβ), the primary amyloid component in extracellular plaques. This review focuses on the current status of Aβ antibodies in clinical development, including their characteristics and challenges that came up in clinical trials with these new biological entities (NBEs). Emphasis is placed on the current view of common side effects observed with passive immunotherapy, so-called amyloid-related imaging abnormalities (ARIAs), and potential ways to overcome this issue. Among these new ideas, a special focus is placed on molecules that are directed against post-translationally modified variants of the Aβ peptide, an emerging approach for development of new antibody molecules.

## 1. Introduction

Alzheimer’s Disease (AD) is the most common neurodegenerative disorder worldwide, currently affecting about 40 million people [1,2]. The patient number will prospectively triple in the decades to come [1,2,3]. It is estimated that three out of four dementia cases are characterized by AD-typical pathological changes [3,4]. Despite significant efforts over the last two decades, there are only symptomatic and transiently active treatments available, making AD one of the largest unmet medical needs. The currently approved symptomatic treatments target neurotransmitter function by inhibiting cholinesterase or antagonizing NMDA receptors. Approved drugs are donepezil, galantamine, rivastigmine (all acetylcholinesterase (AChE) inhibitors), memantine (NMDA receptor antagonist), and a combination of donepezil and memantine [5]. A fourth cholinesterase inhibitor, tacrine, was discontinued in 2013 due to hepatotoxicity, probably related to the production of toxic intermediates (https://www.livertox.nih.gov/Tacrine.htm). Numerous other drugs are currently under investigation. In 2017, 105 different new molecular entities (NMEs) were in clinical development for the indication of AD. The majority (70%) address potential disease-modifying therapies (DMTs) to slow or reverse the progression of AD [6]. The small molecule trials address a variety of processes, including anti-oxidants [7], PPAR agonists [8], monoamine oxidase inhibitors [9], and BACE-inhibitors [10].

AD is characterized by two histopathological hallmarks: the deposition of the amyloid β (Aβ) peptide within plaques and the brain vasculature, and intracellular aggregation of the hyperphosphorylated protein tau in neurofibrillary tangles [11,12,13]. There is compelling evidence that the accumulation of Aβ precedes the spreading of tau pathology, brain structural changes, and symptomatic changes by years if not decades [14]. Moreover, a small proportion of AD cases are caused by autosomal dominant mutations in the amyloid precursor protein (APP), presenilin 1 (PS1) or presenilin 2 (PS2) genes. The gene products are involved in the formation of the Aβ peptide. The resulting influence ranges from increased Aβ production, overproportioned formation of species with a high aggregation propensity, or influence on the compartment in which APP is processed [15,16,17]. Protective mutations have also been described, which lead to reduced cleavage of APP and thus the lowering of Aβ production and the risk for development of dementia [18]. The association of the formation of Aβ with inherited early-onset AD (EOAD) resulted in the amyloid hypothesis of Alzheimer’s disease. According to the hypothesis, Aβ in its aggregated form represents the central trigger for a cascade of pathophysiological brain changes, eliciting tau hyperphosphorylation, neuronal damage, synapse and cell loss, and dementia [16]. Although substantially supported by novel amyloid imaging techniques and these inherited AD cases, the hypothesis has been the subject of much debate for years. This was caused by obstacles in drug and concept design and numerous failures of drugs that were designed to address the formation and/or accumulation of the Aβ molecule. Several reasons might account for these failures, such as low selectivity of small molecule inhibitors (e.g., for γ-secretase) [19], and inefficient penetration of the blood–brain barrier, which initially complicated the development of BACE1-inhibitors [20,21]. However, the primary reason might be due to the inclusion of non-AD dementia patients in clinical trials and the late start of treatment within the course of the disease [22,23]. Therefore, current clinical trials recruit only patients showing a clear AD signature (e.g., by imaging or biomarkers), and start treatment of patients with prodromal to early AD [22]. The failures of two monoclonal antibodies in clinical phase III—bapineuzumab and solanezumab [24,25]—contributed to the questioning of the amyloid hypothesis as a basic target for intervention. However, the critical assessment of these failures led to the development of new antibody molecules and strategies for their application. This review summarizes the state of the development of these antibodies, and strategies and challenges for their development. Among these molecules, the monoclonal antibody aducanumab has been shown to significantly reduce amyloid load and to halt cognitive decline in two cognitive measures over a treatment period of 54 weeks in a Phase 1b study [26], thus providing a strong argument in favor of the amyloid hypothesis.

## 2. Monoclonal Antibodies Targeting Aβ in Clinical Development

As shown in Table 1, there are four different antibodies currently being tested in clinical phase III studies: solanezumab, aducanumab, gantenerumab, and crenezumab. Although all of these antibodies recognize Aβ, they differ significantly with regard to their origin and their selectivity to aggregated forms (i.e., oligomers and fibrils). Solanezumab and crenezumab bind to epitopes within the mid-region of Aβ, which is a region that undergoes a structural change to form an intramolecular anti-parallel β-sheet during fibril formation [27,28]. Hence, these antibodies preferably bind to monomeric Aβ like solanezumab [29] or are reported to recognize more specific different forms of oligomers and fibers in case of crenezumab [30]. In contrast, aducanumab and gantenerumab recognize—at least as one primary interaction site—the N-terminal region of Aβ, and are apparently most effective at binding aggregated forms (e.g., fibrils). Another differentiating point for these antibodies regards their stem (fragment crystallizable or Fc) region, which is the invariable part of antibodies which mediates the interaction with immune cells to elicit phagocytosis and degradation of the antigen–antibody complex [31]. There are four classes of human immunoglobulin G (IgG ) molecules differentiated that vary according to the Fc region: IgG1, 2, 3, and 4. These molecules display significantly different features: For instance, in humans IgG1 molecules mediate strong binding to different forms of Fcγ receptors on antigen-presenting cells and strongly elicit the activation of C1q of the classical complement cascade. In stark contrast, IgG2 and IgG4 show only weak interaction with Fcγ receptors and do not mediate complement activation [32]. Notably, the majority of human antibodies under development (Table 1) are IgG1 derivatives. This may be somewhat surprising, as this subtype also induces the release of pro-inflammatory cytokines, which might contribute to the observed side effects such as amyloid-related imaging abnormalities (ARIAs). A very prominent example for that is provided by a phase 1b study of aducanumab [26]. Here, a dose-dependent reduction of the amyloid load as measured by positron emission tomography (PET) was observed over a one-year treatment period. More importantly, the treatment also resulted in a significant cognitive stabilization in mini-mental status examination (MMSE) and clinical dementia rating sum of boxes (CDR-SB) test paradigms at the high dose of 10 mg/kg. Although the study has shown for the first time efficacy of amyloid immunotherapy and therefore provides a prominent proof of the amyloid cascade hypothesis, there was a significant liability for development of ARIA with edema (ARIA-E) in the high-dose group. More than one-third of all treated patients developed imaging abnormalities. In contrast, crenezumab, an IgG4 antibody, did not show side effects up to doses of 60 mg/kg [33]. In order to avoid this side effect, several antibodies in clinical development have been modified to attenuate their effector function. For instance, MEDI1814 and GSK93776, which recognize the Aβ C- or N-terminus, respectively, represent IgG1 molecules that harbor mutations to reduce the effector function. Although the switch of the antibody subtype to lower antibody-dependent cell-mediated cytotoxicity (ADCC) and Complement-dependent cytotoxicity (CDC) appears conceivable in terms of the prevention of side effects, there has also been failure reported with such modified molecules. The most prominent example is the antibody ponezumab, whose development was discontinued after Phase 1 clinical studies. The antibody was of an IgG2 subtype, and therefore ADCC and CDC functions were significantly lower compared to IgG1 antibodies. The Phase 1 data suggested the entry of the antibody into the brain and an accumulation of the antibody and presumably antigen–antibody complexes within the circulation. This observation raised safety concerns and finally led to discontinuation of its development.

## 3. Tackling Current Limitation—ARIA and BBB Penetration

In light of the promising Phase1b results of aducanumab, the most important challenge for the development of antibodies might be devising ways to prevent imaging abnormalities such as ARIA-E and ARIA with hemorrhage (ARIA-H). The appearance of these side effects seems to be closely related to the amyloid burden in the vasculature of the patients [54]. Vascular amyloid is characterized by deposition of Aβ peptides within the vessel wall of arterial brain vessels. This phenomenon is not restricted to Alzheimer’s disease. Other amyloidoses are characterized by cerebral amyloid angiopathy (CAA), such as familial Danish dementia. The appearance of CAA in sporadic Alzheimer’s disease is linked to the ApoE4 allele, but also mutations within the Aβ molecule predispose to the deposition of Aβ within the vessels and, occasionally, hemorrhage [55,56].

The presence of vascular amyloid generates a challenge for the development of immunotherapy. Because the antibodies bind to the vascular deposits, monocytes and other lymphocytes are recruited, which are stimulated to clear the amyloid. The binding of the antibody complexes to the Fc receptors (CD16, CD64, and CD32) on macrophage-like cells stimulates the expression of proteases, and in turn, the degradation of extracellular matrix. As a consequence, the barrier function of the vessel wall is weakened and interstitial fluid may enter the brain tissue, which is observed as ARIA-E. In cases of more severe damage to the vessels, hemorrhages might result. As mentioned in the previous section, the antibodies differ according to their subclass and to the effector function. Typically, the IgG1 subtypes strongly stimulate phagocytosing cells and are therefore more prone to ARIA-E side effects. Based on these observations, different options might exist to preserve the tissue from ARIAs. The first strategy is a slow, progressive increase in dosing. Other studies have shown that the Aβ from vessels is cleared first, before parenchymal amyloid is degraded. Therefore, to start with a lower dose might help to decrease the vascular burden first, and after successful initial treatment, higher doses are reached to clear brain parenchyma of deposits. A second strategy to attenuate the effector function of the antibodies is to reduce the binding to Fc receptors and in turn, vessel wall damage by the activated lymphocytes. A third strategy might be to target specific Aβ epitopes, which are not present or are underrepresented in vascular amyloid compared with parenchymal Aβ. Among these is MEDI 1814, which is specific to the Aβ 42C-terminus, accordingly sparing Aβ40 peptides. Because Aβ42 is underrepresented in vascular amyloid [57,58,59], this might help to reduce antibody binding to the vessels. Similarly, another strategy is to target modifications of Aβ, which will be discussed below.

Finally, an improved passage of antibodies through the blood–brain barrier might help to reduce dosage, and thus reduce common side effects. Different ways of achieving this have been discovered or are currently under evaluation, either by modification of the protein drug molecule or by influencing the blood–brain barrier. Prominent ways to modify the antibody molecule are based on targeting receptor molecules at the epithelial cells. Typically, these receptors are shuttling cargo between the blood and the brain parenchyma and are therefore predestined for the improvement of antibody delivery to the brain. The most prominent receptors are probably the transferrin and insulin receptors [60,61,62,63]. Antibodies developed to target these receptors typically contain one binding site for the receptor and another site(s) for binding of the antigen. The most straightforward case is represented by bispecific IgG molecules composed of two different heavy and light chains. However, there have been numerous derivatives of such molecules described, containing transporter-specific fusions of antibody parts linked to the heavy or light chains of the antibodies [63]. Although these strategies have been described as increasing the brain penetrance of the antibodies significantly, the increase of the concentration appears to be limited by different factors. On the one hand, the affinity of the antibody towards the transporter molecule appears challenging. High affinity does not lead to a significant increase of the antibody in the brain, because the antibody is only weakly released after binding. A low affinity results in inefficient binding on the epithelium, and thus low transport levels. Therefore, an optimal affinity needs to be reached, which in turn, apparently limits the efficiency of the transport [64,65,66]. Another reason why such antibodies have not yet reached a clinical stage is that the binding of the receptors might lead to downregulation of these transporters under chronic treatment, and thus increase the risk of side effects and loss of antibody efficacy.

These shortcomings have potentially also led to other techniques which are currently being explored to increase the efficiency of immunotherapy. Focused ultrasound (FUS) just recently provided very promising results to increase the penetration of drugs through the blood–brain barrier (BBB) [67,68]. The method is based on the formation of microbubbles, which due to the application of ultrasound, transiently open the BBB for a limited period of time to allow increased penetration of drugs from the blood into the brain parenchyma. FUS, and a modified version called scanning ultrasound (SUS), have been shown preclinically to increase therapeutic antibody delivery to the brain in several AD-like mouse models [69,70]. The method offers the great advantage of a broad application for different drugs, not limited to antibody molecules.

## 4. Post-Translationally Modified Aβ Peptides: Emerging Targets for Immunotherapy

**Figure 1:**
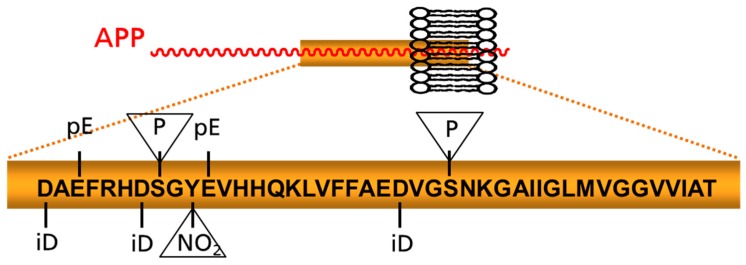
Schematic depiction of prominent posttranslational modifications of Aβ. Modifications appearing within the peptide chain and those addressing amino acid side chains (in triangles) are highlighted. APP: amyloid precursor protein; iD: isoaspartate; NO_2_: nitration pE: pyroglutamate; P: phosphorylation site.

The proteolytic processing of APP by BACE1 and γ-secretase results primarily in the formation of Aβ peptides of 40, 42, or 38 amino acids in length (i.e., Aβ1-40, Aβ1-42, or Aβ1-38) [71,72]. However, several studies have shown that these species constitute a minor fraction of the Aβ peptides in AD. The majority of Aβ is post-translationally modified by truncation, isomerization, or covalent modification (Figure 1). N-terminal truncated peptides gained considerable interest because of their exceptional toxicity and their abundance in familial AD [73,74,75,76,77]. Among the truncated forms, those beginning at position 3 or 11 and containing an N-terminal pyroglutamate (pGlu) instead of glutamate are certainly the most intensively studied [78]. The total amount of pGlu-Aβ in an AD brain differs among studies—the majority of reports suggest a content of 5–25% [79,80,81,82,83]. The pGlu-Aβ content in the brain has been shown to increase as AD pathology progresses [82,84]. The rise of pGlu-Aβ is accompanied by a decrease of full length-Aβ species, and thus the pGlu-content shows an inverse correlation with the cognitive status [85]. Numerous studies have shown that the N-terminal pGlu-modification increases the aggregation propensity of the amyloid peptides [77,86,87,88]. Moreover, the pGlu-peptides show a rapid formation of oligomers with higher hydrophobicity and neurotoxicity compared to full-length Aβ [77,88]. Co-aggregation studies with full-length Aβ have shown that pGlu-Aβ induces the formation of small molecular weight oligomers, which is apparently caused by molecular priming [77]. On the basis of these characteristics, drug development approaches that address the formation and/or clearance of pGlu-Aβ are underway. These are either based on the inhibition of glutaminyl cyclase (QC) or targeting the pGlu-Aβ peptide by monoclonal antibodies [89,90,91].

With regard to immunotherapy, several studies in transgenic mice have shown the efficacy of anti-pGlu3-Aβ antibodies. We were the first to report that both preventive and therapeutic treatment showed significant reduction of pGlu-Aβ and also total Aβ deposits [92]. The decrease of total amyloid burden by the antibody is noteworthy because transgenic mice display a much lower pGlu3-Aβ burden compared to human AD [76,93]. A later study substantiated these findings in PDAPP mice [40] by demonstrating that a different pGlu3-specific antibody significantly reduced the total amyloid burden in mice significantly, in the absence of microbleeds. A later study by us to determine the effects of pGlu3-Aβ immunotherapy on cognition in AD-like transgenic mice revealed a significant improvement of spatial learning in water T-maze upon treatment. The pGlu3-antibody treatment was superior in this regard, over an antibody specific to the N-terminus of Aβ (3A1) [94]. No increase in the incidence of microbleeds was observed. Notably, we did not observe an increase of Aβ in the plasma of mice that were treated with the pGlu3-Aβ antibody. In contrast, treatment with 3A1 led to a significant increase of the plasma Aβ concentration, suggesting different mechanisms of action of these molecules. Meanwhile, a pGlu3-Aβ antibody, LY3002813, is the first to reach clinical testing (Table 1). The initial results of a phase 1 study were presented at the Alzheimer’s Association International Conference (AAIC) in 2016 [41]. In that study, patients received 0.1 to 10 mg/kg by monthly intravenous injections. Aβ-PET tomography was used to evaluate treatment effects and as an inclusion criterion. Treatment with the 10 mg/kg dose significantly reduced the plaque load in the brain by about 40%. Incidence of ARIA-E was not observed, but two cases of ARIA-H have been reported. Although the amyloid reduction was very impressive, the molecule appeared to elicit a human anti-human antibody response, markedly reducing the half-life of the antibody in circulation. 

On the basis of several studies in mice and these initial clinical findings, antibodies directed against the pGlu3-Aβ peptides appear to be very promising “second-generation” NBEs for the treatment of AD. Monoclonal antibodies targeting modified Aβ offer some unique and potentially advantageous characteristics: (1) The antibodies directly target a disease-specific form of Aβ with high neurotoxic potential; (2) As a physiological function of Aβ1-40/42 has been suggested by numerous studies [95,96,97,98], specific targeting of pGlu-Aβ should not interfere with that role; (3) The pGlu-modification prominently alters the structure of the Aβ molecule, enabling efficient isolation of antibodies with low cross-reactivity with other Aβ entities and the amyloid precursor protein, APP [92]. Moreover, the targeting of modified Aβ offers some technical advantages with regard to target engagement and, potentially, side effects. Because the pGlu-modified peptides are only distributed to aggregates within the brain and are absent in plasma, the antibodies are not sequestered within the periphery by the target molecule [94]. This potentially enables better brain distribution of the drug and, as has been shown for pGlu-Aβ antibodies, no increase of Aβ within the plasma as observed with other antibodies [94]. The specific targeting of one modification might also present a rationale for the low incidence of ARIA or microhemorrhage observed in preclinical and clinical studies. Because the density of the pGlu-Aβ epitopes is low compared with general Aβ epitopes within CAA, the recruitment of microglia or other immune cells to these immune complexes to the vasculature is potentially lower, and the appearance of edema is reduced as observed with LY3002813.

Although the pGlu-modified Aβ represents the best studied N-terminally modified species so far and the pGlu3-Aβ antibodies are by far the most advanced in development, other modifications might also be targeted in future studies. For instance, numerous studies point toward other modifications, which potentially increase the aggregation propensity and toxicity of Aβ. Among those, phosphorylation at position 8 and 23 or nitration at position 10 are potentially interesting targets for monoclonal antibodies [99,100,101,102]. Thus, it remains to be seen whether targeting of modified Aβ per se offers the above-described potential advantages or whether these are only observed with protein drugs targeting pGlu3-Aβ.

## 5. Conclusions

Aβ immunotherapeutics are among the most advanced drugs in clinical development for the treatment of AD. In spite of several failures of monoclonal antibodies in late-stage clinical studies, these trials have provided an invaluable gain in the understanding of the disease mechanism, supporting the amyloid cascade hypothesis. The first generation of protein drugs still leave significant room for improvement regarding the prevention of side effects and the penetration of the blood–brain barrier (BBB). The improvement of BBB penetration, tailored antibody specificity, and affinity might also finally help to overcome the drawbacks of treatment cost typically associated with passive immunotherapy. In addition, current and future approaches to test combination therapy with Aβ-directed small-molecule drugs promisingly appear to be more effective, safer, and less costly.

## Figures and Tables

**Table 1:** Overview of amyloid β (Aβ)-directed antibodies being tested in clinical trials. Further information can be found on https://clinicaltrials.gov/. PI, PII, and PIII refer to phases one, two, and three of clinical trials, respectively. AD: Alzheimer’s disease; ARIA: amyloid-related imaging abnormality; FAD: Familial Alzheimer’s Disease; IV: intravenous; SC: subcutaneous.

Antibody/IgG Subtype	Company	Specificity	Dosage	Development Stage
**Bapineuzumab**/IgG1 AAB-001 (humanized mouse 3D6)	Janssen/Pfizer	Aβ 1–5 (helical, N-terminal D sensitive)	PI: 12-month 0.5, 1.5, or 5 mg/kg PII: 18-month 0.15, 0.5, 1, or 2 mg/kg PIII: 18-month 0.5 mg/kg 1.0 mg/kg	Terminated in August 2012, because 2 large Phase 3 studies showed no clinical benefit. This decision was not based on any new safety concerns [24,34]
**AAB-003** (PF-05236812) humanized and IgG1 Fc-engineered (Effector function reduced variant of bapineuzumab)	Janssen/Pfizer	Aβ 1–5 (helical, N-terminal D sensitive)	PI: 0.5, 1, 2, 4, 8 mg/kg	Completed 2016, lower toxicity (ARIAs) compared to Bapineuzumab was expected, continuation as open-label extension study to February 2017 [35], discontinued in January 2018 by Pfizer.
**Ponezumab** (PF-04360365, RN1219) IgG2 (humanized mouse monoclonal antibody)	Pfizer (developed by Rinat Nsc.)	binds the free carboxy-terminal amino acids 33–40 of Aβ 1–40	PII 10 mg/kg 2009–2011 PII 8.5 mg/kg 2008–2011	Nov 2011, Pfizer Inc. discontinued development of ponezumab [36,37]
**Solanezumab** (LY2062430) IgG1 (humanized mouse 266])	Eli Lilly	Aβ 16–26 accessible only on monomeric Aβ	PIII 2009–2012EXPEDITION PIII 2010–2014 EXPEDITION EXT PIII 2013–2016 EXPEDIRION 3 PIII 2016–2017 EXPEDITION PRO (solanezumab 400 milligrams (mg) every 4 weeks for 76 weeks PIII A4 (2014–2022) 400–1600 mg IV every 4 weeks for 240 weeks	Failed in 2012 in primary endpoint and terminated in May 2017. Insufficient scientific evidence that solanezumab would likely demonstrate a meaningful benefit to patients with prodromal AD as defined by the study protocol [25,38]. Active in FAD PIII DIAN-TU (2012–2023) [39] Active in PIII A4 study in older individuals at risk for AD (2014–2022)
**LY3002813** IgG1 (humanized mouse mE8-IgG2a)	Eli Lilly	pE3-Aβ	0.1 mg/kg to 10 mg/kg, infused monthly up to four times, and a single subcutaneous injection against placebo for safety	PI 2017–2020 No cases of ARIA were seen in this small trial, but there were two asymptomatic cases of ARIA-H (hemorrhage). The antibody was reported to be strongly immunogenic [40,41]. PII 2017–2020 in combination with BACE inhibitor LY3202626 in early symptomatic AD (ClinicalTrials.gov Identifier: NCT03367403)
**Gantenerumab** (RG1450, RO4909832) IgG1 (full human)	Hoffmann-La Roche	Aβ 2–5 (−9) + 23–25 bind with subnanomolar affinity to a conformational epitope on Aβ fibrils. It binds both N-terminal and central amino acids of Aβ	PIII 225 mg SC 2010–2019 FAD PIII DIAN-TU (2012–2023) two new PI trials started 2016, investigating subcutaneous administration of higher doses of gantenerumab.	PIII active On March 6, 2017, MorphoSys, which partners in the development of gantenerumab, announced Roche will start two new Phase 3 trials of the immunotherapy for prodromal AD in 2017 [42].
**Crenezumab** (RG7412, MABT5102A) IgG4 (humanized mouse MABT5102)	Genentech 8 discov. by AC immune)	Aβ 13–24 (conformational epitopes?) Binds fibrillar, oligomeric, and monomeric Abeta.	PIII CRED 2016–2020 [21] up to 60 mg/kg PII (2013–2022) SC (every 2 weeks) or IV (every 4 weeks) for at least 260 weeks	PIII CREAD 2 2017–2022 [33,43] Prodromal to mild AD; mild to moderate AD (2 different trials) PII FAD PSEN1 E280A autosomal-dominant mutation carriers
**BAN2401** IgG1 (humanized mAb158)	Eisai (discov. by BioArctic) 2014 coll. with Biogen	recognizes Aβ protofibrils	two PI: 2.5, 5 and 10 mg/kg PII: 2.5, 5 and 10 mg/kg	PII 2012–2018 patients with early AD [44,45,46]
**Aducanumab** IgG1 (BIIB037/BART full human)	Biogen (discov. by Neuri-Mmune)	recognizes Aβ oligomer and fibrils	PIb PRIME PIII ENGAGE 2015–2022 dosage unknown PIII EMERGE 2015–2022 dosage unknown	PIII in prodromal AD patients [26,47,48]
**SAR228810** (humanized Ab 13C3); IgG4 (like) framework	Sanofi	recognizes Aβ protofibrils	PI 2012–2015 study to assess the safety and the concentration-time profile with IV and SC injection	PI [49]
**MEDI1814** IgG1 3x mut ADCC-, CDC-	AstraZeneca	selective for Aβ42 C-terminus	PI 2014–2016 (2017) for IV and SC injection; 25 to 1800 mg total	PI [50]
**GSK933776** (humanized, IgG1 reduced in ADCC and CDC)	GlaxoSmithKline	against the N-terminus of the Aβ	PI: 1, 3, or 6 mg/kg	No further development in AD. In 2015, this antibody was in PII for retinal amyloidosis in connection with dry age-related macular degeneration (dry AMD) [51,52]
RN6G (Pf-04382923) IgG2 (humanized mouse monoclonal antibody)	Pfizer (dev. By Rinat Neuroscience)	C-terminus of Aβ, no differentiation between 40/42	PII 2012–2013 IV injection 2.5 mg/kg up to a maximum of 15 mg/kg	PII Developed in dry AMD [53]

## References

[1] Prince M., Bryce R., Albanese E., Wimo A., Ribeiro W., Ferri C.P. (2013). The global prevalence of dementia: A systematic review and metaanalysis. Alzheimer’s Dement..

[2] Prince M., Ali G.C., Guerchet M., Prina A.M., Albanese E., Wu Y.T. (2016). Recent global trends in the prevalence and incidence of dementia, and survival with dementia. Alzheimer’s Res. Ther..

[3] Burns A., Iliffe S. (2009). Dementia. BMJ.

[4] Burns A., Iliffe S. (2009). Alzheimer’s disease. BMJ.

[5] Glynn-Servedio B.E., Ranola T.S. (2017). AChE Inhibitors and NMDA Receptor Antagonists in Advanced Alzheimer’s Disease. Consult. Pharm..

[6] Cummings J., Lee G., Mortsdorf T., Ritter A., Zhong K. (2017). Alzheimer’s disease drug development pipeline: 2017. Alzheimer’s Dement..

[7] Onyango I.G. (2018). Modulation of mitochondrial bioenergetics as a therapeutic strategy in Alzheimer’s disease. Neural Regen. Res..

[8] Cheng H., Shang Y., Jiang L., Shi T.L., Wang L. (2016). The peroxisome proliferators activated receptor-gamma agonists as therapeutics for the treatment of Alzheimer’s disease and mild-to-moderate Alzheimer’s disease: A meta-analysis. Int. J. Neurosci..

[9] Cai Z. (2014). Monoamine oxidase inhibitors: Promising therapeutic agents for Alzheimer’s disease. Mol. Med. Rep..

[10] Timmers M., Sinha V., Darpo B., Smith B., Brown R., Xue H., Ferber G., Streffer J., Russu A., Tritsmans L. (2018). Evaluating Potential QT Effects of JNJ-54861911, a BACE Inhibitor in Single- and Multiple-Ascending Dose Studies, and a Thorough QT Trial with Additional Retrospective Confirmation, Using Concentration-QTc Analysis. J. Clin. Pharmacol..

[11] Jack C.R., Knopman D.S., Jagust W.J., Shaw L.M., Aisen P.S., Weiner M.W., Petersen R.C., Trojanowski J.Q. (2010). Hypothetical model of dynamic biomarkers of the Alzheimer’s pathological cascade. Lancet Neurol..

[12] Querfurth H.W., LaFerla F.M. (2010). Alzheimer’s disease. N. Engl. J. Med..

[13] Hardy J., Selkoe D.J. (2002). The amyloid hypothesis of Alzheimer’s disease: Progress and problems on the road to therapeutics. Science.

[14] Villemagne V.L., Burnham S., Bourgeat P., Brown B., Ellis K.A., Salvado O., Szoeke C., Macaulay S.L., Martins R., Maruff P. (2013). Amyloid beta deposition, neurodegeneration, and cognitive decline in sporadic Alzheimer’s disease: A prospective cohort study. Lancet Neurol..

[15] Citron M., Westaway D., Xia W., Carlson G., Diehl T., Levesque G., Johnson W., Lee M., Seubert P., Davis A. (1997). Mutant presenilins of Alzheimer’s disease increase production of 42-residue amyloid beta-protein in both transfected cells and transgenic mice. Nat. Med..

[16] Selkoe D.J., Hardy J. (2016). The amyloid hypothesis of Alzheimer’s disease at 25 years. EMBO Mol. Med..

[17] Jayne T., Newman M., Verdile G., Sutherland G., Munch G., Musgrave I., Moussavi Nik S.H., Lardelli M. (2016). Evidence For and Against a Pathogenic Role of Reduced gamma-Secretase Activity in Familial Alzheimer’s Disease. J. Alzheimer’s Dis..

[18] Jonsson T., Atwal J.K., Steinberg S., Snaedal J., Jonsson P.V., Bjornsson S., Stefansson H., Sulem P., Gudbjartsson D., Maloney J. (2012). A mutation in APP protects against Alzheimer’s disease and age-related cognitive decline. Nature.

[19] Extance A. (2010). Alzheimer’s failure raises questions about disease-modifying strategies. Nat. Rev. Drug Discov..

[20] Carter M.D., Simms G.A., Weaver D.F. (2010). The development of new therapeutics for Alzheimer’s disease. Clin. Pharmacol. Ther..

[21] De Strooper B. (2010). Proteases and proteolysis in Alzheimer disease: A multifactorial view on the disease process. Physiol. Rev..

[22] Schneider L.S., Mangialasche F., Andreasen N., Feldman H., Giacobini E., Jones R., Mantua V., Mecocci P., Pani L., Winblad B. (2014). Clinical trials and late-stage drug development for Alzheimer’s disease: An appraisal from 1984 to 2014. J. Intern. Med..

[23] Giacobini E., Gold G. (2013). Alzheimer disease therapy—Moving from amyloid-beta to tau. Nat. Rev. Neurol..

[24] Vandenberghe R., Rinne J.O., Boada M., Katayama S., Scheltens P., Vellas B., Tuchman M., Gass A., Fiebach J.B., Hill D. (2016). Bapineuzumab for mild to moderate Alzheimer’s disease in two global, randomized, phase 3 trials. Alzheimer’s Res. Ther..

[25] Honig L.S., Vellas B., Woodward M., Boada M., Bullock R., Borrie M., Hager K., Andreasen N., Scarpini E., Liu-Seifert H. (2018). Trial of Solanezumab for Mild Dementia Due to Alzheimer’s Disease. N. Engl. J. Med..

[26] Sevigny J., Chiao P., Bussiere T., Weinreb P.H., Williams L., Maier M., Dunstan R., Salloway S., Chen T., Ling Y. (2016). The antibody aducanumab reduces Abeta plaques in Alzheimer’s disease. Nature.

[27] Gremer L., Scholzel D., Schenk C., Reinartz E., Labahn J., Ravelli R.B.G., Tusche M., Lopez-Iglesias C., Hoyer W., Heise H. (2017). Fibril structure of amyloid-beta(1-42) by cryo-electron microscopy. Science.

[28] Zhao J., Nussinov R., Ma B. (2017). Mechanisms of recognition of amyloid-beta (Abeta) monomer, oligomer, and fibril by homologous antibodies. J. Biol. Chem..

[29] Crespi G.A., Hermans S.J., Parker M.W., Miles L.A. (2015). Molecular basis for mid-region amyloid-beta capture by leading Alzheimer’s disease immunotherapies. Sci. Rep..

[30] Ultsch M., Li B., Maurer T., Mathieu M., Adolfsson O., Muhs A., Pfeifer A., Pihlgren M., Bainbridge T.W., Reichelt M. (2016). Structure of Crenezumab Complex with Abeta Shows Loss of beta-Hairpin. Sci. Rep..

[31] Eggleton P., Javed M., Pulavar D., Sheldon G. (2015). Immune Complexes.

[32] Vidarsson G., Dekkers G., Rispens T. (2014). IgG subclasses and allotypes: From structure to effector functions. Front. Immunol..

[33] Blaettler T., Smith J., Smith J., Paul R., Asnaghi V., Fuji R., Quartino A., Honigberg L., Rabbia M., Yule S. (2016). Clinical Trial Design of CREAD: A Randomized, Double-Blind, Placebo-Controlled, Parallel-Group Phase 3 Study to Evaluate Crenezumab Treatment in Patients with Prodromal-to-Mild Alzheimer’s Disease. Alzheimer’s Dement..

[34] Lacey L., Bobula J., Rudell K., Alvir J., Leibman C. (2015). Quality of Life and Utility Measurement in a Large Clinical Trial Sample of Patients with Mild to Moderate Alzheimer’s Disease: Determinants and Level of Changes Observed. Value Health.

[35] Delnomdedieu M., Duvvuri S., Li D.J., Atassi N., Lu M., Brashear H.R., Liu E., Ness S., Kupiec J.W. (2016). First-In-Human safety and long-term exposure data for AAB-003 (PF-05236812) and biomarkers after intravenous infusions of escalating doses in patients with mild to moderate Alzheimer’s disease. Alzheimer’s Res. Ther..

[36] Burstein A.H., Zhao Q., Ross J., Styren S., Landen J.W., Ma W.W., McCush F., Alvey C., Kupiec J.W., Bednar M.M. (2013). Safety and pharmacology of ponezumab (PF-04360365) after a single 10-minute intravenous infusion in subjects with mild to moderate Alzheimer disease. Clin. Neuropharmacol..

[37] Landen J.W., Zhao Q., Cohen S., Borrie M., Woodward M., Billing C.B., Bales K., Alvey C., McCush F., Yang J. (2013). Safety and pharmacology of a single intravenous dose of ponezumab in subjects with mild-to-moderate Alzheimer disease: A phase I, randomized, placebo-controlled, double-blind, dose-escalation study. Clin. Neuropharmacol..

[38] Siemers E.R., Sundell K.L., Carlson C., Case M., Sethuraman G., Liu-Seifert H., Dowsett S.A., Pontecorvo M.J., Dean R.A., DeMattos R. (2016). Phase 3 solanezumab trials: Secondary outcomes in mild Alzheimer’s disease patients. Alzheimer’s Dement..

[39] Bateman R.J., Benzinger T.L., Berry S., Clifford D.B., Duggan C., Fagan A.M., Fanning K., Farlow M.R., Hassenstab J., McDade E.M. (2017). The DIAN-TU Next Generation Alzheimer’s prevention trial: Adaptive design and disease progression model. Alzheimer’s Dement..

[40] Demattos R.B., Lu J., Tang Y., Racke M.M., Delong C.A., Tzaferis J.A., Hole J.T., Forster B.M., McDonnell P.C., Liu F. (2012). A plaque-specific antibody clears existing beta-amyloid plaques in Alzheimer’s disease mice. Neuron.

[41] Irizarry M., Sims J., Lowe S., Nakano M., Hawdon A., Willis B., Gonzales C., Liu P., Fujimoto S., Dean R. (2016). Safety, Pharmacokinetics [PK], and Florbetapir F-18 Positron Emission Tomography [PET] after Multiple Dose Administration of LY3002813, a ß-Amyloid Plaque-Specific Antibody, in Alzheimer’s Disease [AD]. Alzheimer’s Dement..

[42] Bohrmann B., Baumann K., Benz J., Gerber F., Huber W., Knoflach F., Messer J., Oroszlan K., Rauchenberger R., Richter W.F. (2012). Gantenerumab: A novel human anti-Abeta antibody demonstrates sustained cerebral amyloid-beta binding and elicits cell-mediated removal of human amyloid-beta. J. Alzheimer’s Dis..

[43] Adolfsson O., Pihlgren M., Toni N., Varisco Y., Buccarello A.L., Antoniello K., Lohmann S., Piorkowska K., Gafner V., Atwal J.K. (2012). An effector-reduced anti-beta-amyloid (Abeta) antibody with unique abeta binding properties promotes neuroprotection and glial engulfment of Abeta. J. Neurosci..

[44] Tucker S., Moller C., Tegerstedt K., Lord A., Laudon H., Sjodahl J., Soderberg L., Spens E., Sahlin C., Waara E.R. (2015). The murine version of BAN2401 (mAb158) selectively reduces amyloid-beta protofibrils in brain and cerebrospinal fluid of tg-ArcSwe mice. J. Alzheimer’s Dis..

[45] Logovinsky V., Satlin A., Lai R., Swanson C., Kaplow J., Osswald G., Basun H., Lannfelt L. (2016). Safety and tolerability of BAN2401—A clinical study in Alzheimer’s disease with a protofibril selective Abeta antibody. Alzheimer’s Res. Ther..

[46] Satlin A., Wang J., Logovinsky V., Berry S., Swanson C., Dhadda S., Berry D.A. (2016). Design of a Bayesian adaptive phase 2 proof-of-concept trial for BAN2401, a putative disease-modifying monoclonal antibody for the treatment of Alzheimer’s disease. Alzheimer’s Dement..

[47] Kastanenka K.V., Bussiere T., Shakerdge N., Qian F., Weinreb P.H., Rhodes K., Bacskai B.J. (2016). Immunotherapy with Aducanumab Restores Calcium Homeostasis in Tg2576 Mice. J. Neurosci..

[48] Budd H.S., O’Gorman J., Chiao P., Bussiere T., von Rosenstiel P., Tian Y., Zhu Y., von Hehn C., Gheuens S., Skordos L. (2017). Clinical Development of Aducanumab, an Anti-Abeta Human Monoclonal Antibody Being Investigated for the Treatment of Early Alzheimer’s Disease. J. Prev. Alzheimer’s Dis..

[49] Pradier L., Blanchard V., Debeir T., Barneoud P., Canton T., Menager J., Bohme A., Rooney A., Guillet M., Cameron B. (2013). SAR228810: An antiprotofibrillar beta-amyloid antibody designed to reduce risk of amyloid-related imaging abnormalities [ARIA]. Alzheimer’s Dement..

[50] Bogstedt A., Groves M., Tan K., Narwal R., McFarlane M., Hoglund K. (2015). Development of Immunoassays for the Quantitative Assessment of Amyloid-beta in the Presence of Therapeutic Antibody: Application to Pre-Clinical Studies. J. Alzheimer’s Dis..

[51] Singer M. (2014). Advances in the management of macular degeneration. F1000Prime Rep..

[52] Leyhe T., Andreasen N., Simeoni M., Reich A., von Arnim C.A., Tong X., Yeo A., Khan S., Loercher A., Chalker M. (2014). Modulation of beta-amyloid by a single dose of GSK933776 in patients with mild Alzheimer’s disease: A phase I study. Alzheimer’s Res. Ther..

[53] Ding J.D., Johnson L.V., Herrmann R., Farsiu S., Smith S.G., Groelle M., Mace B.E., Sullivan P., Jamison J.A., Kelly U. (2011). Anti-amyloid therapy protects against retinal pigmented epithelium damage and vision loss in a model of age-related macular degeneration. Proc. Natl. Acad. Sci. USA.

[54] Sperling R.A., Jack C.R., Black S.E., Frosch M.P., Greenberg S.M., Hyman B.T., Scheltens P., Carrillo M.C., Thies W., Bednar M.M. (2011). Amyloid-related imaging abnormalities in amyloid-modifying therapeutic trials: Recommendations from the Alzheimer’s Association Research Roundtable Workgroup. Alzheimer’s Dement..

[55] Sperling R., Salloway S., Brooks D.J., Tampieri D., Barakos J., Fox N.C., Raskind M., Sabbagh M., Honig L.S., Porsteinsson A.P. (2012). Amyloid-related imaging abnormalities in patients with Alzheimer’s disease treated with bapineuzumab: A retrospective analysis. Lancet Neurol..

[56] Pankiewicz J.E., Sadowski M.J. (2017). APOE genotype and Alzheimer’s immunotherapy. Oncotarget.

[57] Gu L., Guo Z. (2013). Alzheimer’s Abeta42 and Abeta40 peptides form interlaced amyloid fibrils. J. Neurochem..

[58] Miller D.L., Papayannopoulos I.A., Styles J., Bobin S.A., Lin Y.Y., Biemann K., Iqbal K. (1993). Peptide compositions of the cerebrovascular and senile plaque core amyloid deposits of Alzheimer’s disease. Arch. Biochem. Biophys..

[59] Gravina S.A., Ho L., Eckman C.B., Long K.E., Otvos L., Younkin L.H., Suzuki N., Younkin S.G. (1995). Amyloid beta protein (A beta) in Alzheimer’s disease brain. Biochemical and immunocytochemical analysis with antibodies specific for forms ending at A beta 40 or A beta 42. J. Biol. Chem..

[60] Boado R.J., Zhang Y., Zhang Y., Xia C.F., Pardridge W.M. (2007). Fusion antibody for Alzheimer’s disease with bidirectional transport across the blood-brain barrier and abeta fibril disaggregation. Bioconjug. Chem..

[61] Pardridge W.M. (2016). Re-engineering therapeutic antibodies for Alzheimer’s disease as blood-brain barrier penetrating bi-specific antibodies. Expert Opin. Biol. Ther..

[62] Hultqvist G., Syvanen S., Fang X.T., Lannfelt L., Sehlin D. (2017). Bivalent Brain Shuttle Increases Antibody Uptake by Monovalent Binding to the Transferrin Receptor. Theranostics.

[63] Niewoehner J., Bohrmann B., Collin L., Urich E., Sade H., Maier P., Rueger P., Stracke J.O., Lau W., Tissot A.C. (2014). Increased brain penetration and potency of a therapeutic antibody using a monovalent molecular shuttle. Neuron.

[64] Yu Y.J., Zhang Y., Kenrick M., Hoyte K., Luk W., Lu Y., Atwal J., Elliott J.M., Prabhu S., Watts R.J. (2011). Boosting brain uptake of a therapeutic antibody by reducing its affinity for a transcytosis target. Sci. Transl. Med..

[65] Couch J.A., Yu Y.J., Zhang Y., Tarrant J.M., Fuji R.N., Meilandt W.J., Solanoy H., Tong R.K., Hoyte K., Luk W. (2013). Addressing safety liabilities of TfR bispecific antibodies that cross the blood-brain barrier. Sci. Transl. Med..

[66] Yu Y.J., Atwal J.K., Zhang Y., Tong R.K., Wildsmith K.R., Tan C., Bien-Ly N., Hersom M., Maloney J.A., Meilandt W.J. (2014). Therapeutic bispecific antibodies cross the blood-brain barrier in nonhuman primates. Sci. Transl. Med..

[67] Parrish K.E., Sarkaria J.N., Elmquist W.F. (2015). Improving drug delivery to primary and metastatic brain tumors: Strategies to overcome the blood-brain barrier. Clin. Pharmacol. Ther..

[68] Krishna V., Sammartino F., Rezai A. (2018). A Review of the Current Therapies, Challenges, and Future Directions of Transcranial Focused Ultrasound Technology: Advances in Diagnosis and Treatment. JAMA Neurol..

[69] Nisbet R.M., Gotz J. (2018). Amyloid-beta and Tau in Alzheimer’s Disease: Novel Pathomechanisms and Non-Pharmacological Treatment Strategies. J. Alzheimer’s Dis..

[70] Leinenga G., Gotz J. (2018). Safety and Efficacy of Scanning Ultrasound Treatment of Aged APP23 Mice. Front. Neurosci..

[71] Andrew R.J., Kellett K.A., Thinakaran G., Hooper N.M. (2016). A Greek Tragedy: The Growing Complexity of Alzheimer Amyloid Precursor Protein Proteolysis. J. Biol. Chem..

[72] Crump C.J., Johnson D.S., Li Y.M. (2013). Development and mechanism of gamma-secretase modulators for Alzheimer’s disease. Biochemistry.

[73] Kummer M.P., Heneka M.T. (2014). Truncated and modified amyloid-beta species. Alzheimer’s Res. Ther..

[74] Russo C., Schettini G., Saido T.C., Hulette C., Lippa C., Lannfelt L., Ghetti B., Gambetti P., Tabaton M., Teller J.K. (2000). Presenilin-1 mutations in Alzheimer’s disease. Nature.

[75] Miravalle L., Calero M., Takao M., Roher A.E., Ghetti B., Vidal R. (2005). Amino-terminally truncated Abeta peptide species are the main component of cotton wool plaques. Biochemistry.

[76] Kawarabayashi T., Younkin L.H., Saido T.C., Shoji M., Ashe K.H., Younkin S.G. (2001). Age-dependent changes in brain, CSF, and plasma amyloid (beta) protein in the Tg2576 transgenic mouse model of Alzheimer’s disease. J. Neurosci..

[77] Nussbaum J.M., Schilling S., Cynis H., Silva A., Swanson E., Wangsanut T., Tayler K., Wiltgen B., Hatami A., Ronicke R. (2012). Prion-like behaviour and tau-dependent cytotoxicity of pyroglutamylated amyloid-beta. Nature.

[78] Jawhar S., Wirths O., Bayer T.A. (2011). Pyroglutamate amyloid-beta (Abeta): A hatchet man in Alzheimer disease. J. Biol. Chem..

[79] Hosoda R., Saido T.C., Otvos L.J., Arai T., Mann D.M., Lee V.M., Trojanowski J.Q., Iwatsubo T. (1998). Quantification of modified amyloid beta peptides in Alzheimer disease and Down syndrome brains. J. Neuropathol. Exp. Neurol..

[80] Russo C., Saido T.C., DeBusk L.M., Tabaton M., Gambetti P., Teller J.K. (1997). Heterogeneity of water-soluble amyloid beta-peptide in Alzheimer’s disease and Down’s syndrome brains. FEBS Lett..

[81] Kuo Y.M., Emmerling M.R., Woods A.S., Cotter R.J., Roher A.E. (1997). Isolation, chemical characterization, and quantitation of A beta 3-pyroglutamyl peptide from neuritic plaques and vascular amyloid deposits. Biochem. Biophys. Res. Commun..

[82] Guntert A., Dobeli H., Bohrmann B. (2006). High sensitivity analysis of amyloid-beta peptide composition in amyloid deposits from human and PS2APP mouse brain. Neuroscience.

[83] Sergeant N., Bombois S., Ghestem A., Drobecq H., Kostanjevecki V., Missiaen C., Wattez A., David J.P., Vanmechelen E., Sergheraert C. (2003). Truncated beta-amyloid peptide species in pre-clinical Alzheimer’s disease as new targets for the vaccination approach. J. Neurochem..

[84] Rijal U.A., Kosterin I., Kumar S., von Arnim C.A., Yamaguchi H., Fandrich M., Walter J., Thal D.R. (2014). Biochemical stages of amyloid-beta peptide aggregation and accumulation in the human brain and their association with symptomatic and pathologically preclinical Alzheimer’s disease. Brain.

[85] Morawski M., Schilling S., Kreuzberger M., Waniek A., Jager C., Koch B., Cynis H., Kehlen A., Arendt T., Hartlage-Rubsamen M. (2014). Glutaminyl cyclase in human cortex: Correlation with (pGlu)-amyloid-beta load and cognitive decline in Alzheimer’s disease. J. Alzheimer’s Dis..

[86] Schilling S., Lauber T., Schaupp M., Manhart S., Scheel E., Bohm G., Demuth H.U. (2006). On the seeding and oligomerization of pGlu-amyloid peptides (in vitro). Biochemistry.

[87] Schlenzig D., Manhart S., Cinar Y., Kleinschmidt M., Hause G., Willbold D., Funke S.A., Schilling S., Demuth H.U. (2009). Pyroglutamate formation influences solubility and amyloidogenicity of amyloid peptides. Biochemistry.

[88] Schlenzig D., Ronicke R., Cynis H., Ludwig H.H., Scheel E., Reymann K., Saido T., Hause G., Schilling S., Demuth H.U. (2012). N-Terminal pyroglutamate formation of Abeta38 and Abeta40 enforces oligomer formation and potency to disrupt hippocampal long-term potentiation. J. Neurochem..

[89] Schilling S., Zeitschel U., Hoffmann T., Heiser U., Francke M., Kehlen A., Holzer M., Hutter-Paier B., Prokesch M., Windisch M. (2008). Glutaminyl cyclase inhibition attenuates pyroglutamate Abeta and Alzheimer’s disease-like pathology. Nat. Med..

[90] Hoffmann T., Meyer A., Heiser U., Kurat S., Bohme L., Kleinschmidt M., Buhring K.U., Hutter-Paier B., Farcher M., Demuth H.U. (2017). Glutaminyl Cyclase Inhibitor PQ912 Improves Cognition in Mouse Models of Alzheimer’s Disease-Studies on Relation to Effective Target Occupancy. J. Pharmacol. Exp. Ther..

[91] Cynis H., Frost J.L., Crehan H., Lemere C.A. (2016). Immunotherapy targeting pyroglutamate-3 Abeta: Prospects and challenges. Mol. Neurodegener..

[92] Frost J.L., Liu B., Kleinschmidt M., Schilling S., Demuth H.U., Lemere C.A. (2012). Passive immunization against pyroglutamate-3 amyloid-beta reduces plaque burden in Alzheimer-like transgenic mice: A pilot study. Neurodegener. Dis..

[93] Frost J.L., Le K.X., Cynis H., Ekpo E., Kleinschmidt M., Palmour R.M., Ervin F.R., Snigdha S., Cotman C.W., Saido T.C. (2013). Pyroglutamate-3 amyloid-beta deposition in the brains of humans, non-human primates, canines, and Alzheimer disease-like transgenic mouse models. Am. J. Pathol..

[94] Frost J.L., Liu B., Rahfeld J.U., Kleinschmidt M., O’Nuallain B., Le K.X., Lues I., Caldarone B.J., Schilling S., Demuth H.U. (2015). An anti-pyroglutamate-3 Abeta vaccine reduces plaques and improves cognition in APPswe/PS1DeltaE9 mice. Neurobiol. Aging.

[95] Puzzo D., Privitera L., Leznik E., Fa M., Staniszewski A., Palmeri A., Arancio O. (2008). Picomolar amyloid-beta positively modulates synaptic plasticity and memory in hippocampus. J. Neurosci..

[96] Puzzo D., Privitera L., Fa’ M., Staniszewski A., Hashimoto G., Aziz F., Sakurai M., Ribe E.M., Troy C.M., Mercken M. (2011). Endogenous amyloid-beta is necessary for hippocampal synaptic plasticity and memory. Ann. Neurol..

[97] Puzzo D., Arancio O. (2013). Amyloid-beta peptide: Dr. Jekyll or Mr. Hyde?. J. Alzheimer’s Dis..

[98] Soscia S.J., Kirby J.E., Washicosky K.J., Tucker S.M., Ingelsson M., Hyman B., Burton M.A., Goldstein L.E., Duong S., Tanzi R.E. (2010). The Alzheimer’s disease-associated amyloid beta-protein is an antimicrobial peptide. PLoS ONE.

[99] Kumar S., Wirths O., Stuber K., Wunderlich P., Koch P., Theil S., Rezaei-Ghaleh N., Zweckstetter M., Bayer T.A., Brustle O. (2016). Phosphorylation of the amyloid beta-peptide at Ser26 stabilizes oligomeric assembly and increases neurotoxicity. Acta Neuropathol..

[100] Kumar S., Walter J. (2011). Phosphorylation of amyloid beta (Abeta) peptides—A trigger for formation of toxic aggregates in Alzheimer’s disease. Aging.

[101] Kumar S., Rezaei-Ghaleh N., Terwel D., Thal D.R., Richard M., Hoch M., Mc Donald J.M., Wullner U., Glebov K., Heneka M.T. (2011). Extracellular phosphorylation of the amyloid beta-peptide promotes formation of toxic aggregates during the pathogenesis of Alzheimer’s disease. EMBO J..

[102] Kummer M.P., Hermes M., Delekarte A., Hammerschmidt T., Kumar S., Terwel D., Walter J., Pape H.C., Konig S., Roeber S. (2011). Nitration of tyrosine 10 critically enhances amyloid beta aggregation and plaque formation. Neuron.

